# Bogijetong decoction and its active herbal components protect the peripheral nerve from damage caused by taxol or nerve crush

**DOI:** 10.1186/s12906-016-1391-7

**Published:** 2016-10-22

**Authors:** So Hyun Ahn, In Ae Chang, Ki-Joong Kim, Chul-Jung Kim, Uk Namgung, Chung-Sik Cho

**Affiliations:** Department of Oriental Medicine, Daejeon University, Daejeon, 300-716 South Korea

**Keywords:** Bogijetong-decoction, Taxol, Nerve injury, Regeneration, Neuropathy

## Abstract

**Background:**

Bogijetong decoction (BGJTD) is a herbal drug formulation used in the traditional Asian medicine to treat neuropathic insults associated with diabetes and anticancer therapy. To understand the biological basis of BGJTD on protective effects against neuropathy, we investigated physiological and biochemical responses of the sciatic nerves deranged by taxol injection or crush injury in the rats.

**Methods:**

Dissociated Schwann cells and neurons were prepared from the sciatic nerve and dorsal root ganglia (DRG) respectively and were treated with taxol and BGJTD. The sciatic nerve in the rat was injected with taxol or given crush injury. Animals were then administered orally with BGJTD. Effects of BGJTD treatment on cultured cells and in vivo sciatic nerves and DRG tissues were examined by immunofluorescence staining and western blot analysis. Sciatic nerve regeneration was assessed by histological observation using retrograde tracing technique and by behavioral hot plate test. Eighteen different herbal components of BGJTD were divided into 4 subgroups and were used to select herbal drugs that enhanced neurite outgrowth in cultured neurons.

**Results:**

Morphological abnormalities in the sciatic nerve axons and DRG tissue caused by taxol injection were largely improved by BGJTD treatment. BGJTD treatment enhanced neurite outgrowth in cultured DRG neurons and improved Schwann cell survival. Phospho-Erk1/2 levels were elevated by BGJTD administration in the injured- or taxol-injected sciatic nerves. Vimentin phosphorylation catalyzed by cell division cycle 2 (Cdc2) kinase was induced from Schwann cells in the sciatic nerves after taxol injection and crush injury, and phospho-vimentin levels were further upregulated by BGJTD treatment. Retrograde tracing of DiI-labeled DRG sensory neurons revealed growth-promoting activity of BGJTD on axonal regeneration. A drug group (Be) composed of 4 active herbal components which were selected by neurite growth-enhancing activity was as effective as BGJDT for the recovery of thermal sensitivity of the hind paws which had been suppressed by taxol administration.

**Conclusions:**

These data suggest that BGJTD and its active herbal components may protects the peripheral nerve from damage caused by taxol injection and nerve crush.

## Background

Peripheral neuropathy, referring to histological and physiological abnormalities caused by infections, nerve damages, diabetes, anticancer therapy and others [1], generates deficits of sensory and motor functions and affects the quality of life significantly. At cellular levels, structural alterations in peripheral axons and Schwann cells may be related with deficits in myelination and axonal transport that lead to abnormal conduction property of action potential through the axon [2].

Taxol, known to stabilize microtubule assembly, prevents dynamic regulation of spindle components during mitosis and thus has been used as anticancer therapeutic agent [3]. Yet, clinical studies reported that repeated administration of taxol can generate peripheral neuropathy by disrupting microtubule structure in axons [4–6]. Addition of taxol to cultured neurons resulted in decreased neurite outgrowth, and in vivo administration of taxol in rats generated morphological changes of myelinated fibers and degeneration of Schwann cells [7, 8]. Other studies indicated morphometric alterations via abnormal action potential propagation [9]. Interestingly however, in vivo application of taxol at low dose was shown to improve axonal regeneration after spinal cord injury, by stabilizing microtubule structure and reducing glial scarring around the injury cavity [10].

Bogijetong decoction (or Bogijetong-tang; BGJTD) is the herbal prescription that was developed to reduce neuropathy caused by diabetes and anticancer therapy [11, 12]. In our previous studies, BGJTD treatment into the streptozotocin (STZ)-induced diabetes animal model induced Schwann cell responses in terms of increased phosphorylation of vimentin by cell division cycle 2 (Cdc2) and induction of phospho-Erk1/2 and β1 integrin proteins [13]. Also in an animal model given both sciatic nerve injury and taxol treatments, in vivo administration of BGJDT improved the regenerative responses of injured axons in addition to Schwann cell activation as occurred similarly in STZ-injected animals [14]. While these studies suggest that BGJTD may play a role in regulating peripheral neuropathy, overall neuropathic responsiveness to STZ-injected animals is indirect, and the neuropathy caused by combined physical and chemical paradigm may complicate the interpretation of neural responses to BGJTD. Here, to understand the mechanistic basis on how BGJTD is beneficiary for alleviating neuropathy, we investigated the effects of BGJTD treatment on the sciatic nerve which had been either injected taxol or given crush injury. By examining morphological and biochemical responses of peripheral neurons and Schwann cells, we found that BGJTD has a protective function of peripheral neural tissues against neuropathic insults. Noting that BGJTD is composed of as many as 18 different herbal components, we performed additional experiment to select active components that could represent the efficacy of BGJTD. A small decoction group (Be) composed of 4 herbal components showed a similar efficacy in thermal sensitivity of the hind paw as BGJTD administration.

## Methods

### Herbal drug extraction

Dried BGJTD was obtained from Daejeon University Korean Hospital (Daejeon, Korea) where the quality control of herbal drugs was in accordance with the Standard of Korean Pharmacopoeia (ver. 9, Korea Food and Drug Administration, Korea). The procedure of identification of 18 individual drugs was executed by Dr. Chung-Sik Cho at Daejeon University Oriental Hospital (Daejeon, Korea). The voucher specimens were deposited in the Herbarium of Pharmacognosy, Department of Oriental Medicine, Daejeon University (Daejeon, Korea). The key information in herbal drugs is summarized in Table 1. BGJTD was used clinically at Korean Traditional Medicine Hospital of Daejeon University (Daejeon, Korea) and its efficacy was verified [11, 12]. A mixture of dried BGJT was suspended in distilled water for 2 h, boiled for 3 h, and filtered with Whatman filter paper (Grade 1, Whatman Inc, Clifton, NJ, USA) three times. The extract was frozen at −70 °C for 4 h and freeze-dried for 24 h. The yield for BGJTD extract was 24 g for 178 g of the initial raw materials. Purified material was stored at −20 °C and used for experiment after diluting with physiological saline solution (1 mg of extract residue/ml in 0.9 % NaCl solution). For the experiment screening the active components, individual herbal drugs of BGJTD were divided into 4 decoction groups according to the traditional medicinal theory (Table 2). Herbal extracts were prepared similarly as above.

**Table 1:** Sources of BGJTD

Scientific name	Part	Production area	Specimen number	Amount (g)
*Astragalus membranaceus*	Root	Jeongseon, ROK^a^	KNP0011	30
*Panax ginseng C. A. Meyer*	Root	Geumsan, ROK	KNP0038	4
*Angelica gigas*	Root	Jeongseon, ROK	KNP0008	7.5
*Rehmannia glutinosa*	Rhizome	Geumsan, ROK	KNP0087	10
Cnidium officinale Makino	Rhizome	Younghae, ROK	KNP0028	5
Paeonia lactiflora Pall	Root	Younghae, ROK	KNP0085	7.5
*Salvia miltiorrhiza*	Root, rhizome	Sichuan, China	KNP0095	12
*Prunus persica*	Seed	Hebei, China	KNP0087	7.5
*Carthamus tinctorius*	Flower	Xingang, Taiwan	KNP0020	7.5
*Spatholobus suberectus Dunn*	Stem	Jeongseon, ROK	KNP0102	12
*Epimedium Koreanum NAKAI*	Whole	Cheorwon, ROK	KNP0030	10
*Lumbricidae*	Whole	Jiangxi, China	KNP0053	5
*Pueraria thunbergiana*	Root	Younghae, ROK	KNP0089	8
*Pteridium aquilinum var. latiusculum*	Rhizome	Guangdong, China	KNP0023	8
*Albizia julibrissin Durazz*	Peel	Gyeongju, ROK	KNP0007	12
*Uncaria rhynchophylla*	Branch	Hunan, China	KNP0120	12
*Chaenomeles sinensis*	Fruit	Yeongcheon, ROK	KNP0022	8
*Crassostrea gigas*	Shell	Mokpo, ROK	KNP0074	12
Total amount				178

^a^Republic of Korea

**Table 2:** Subgroups of BGJTD

Subgroups	Composition^a^	Applications
Ba	*Astragalus membranaceus* (30), *Panax ginseng C. A.Meyer* (4, ginsenoside Rg1, Rb1)^b^, *Epimedium Koreanum NAKAI* (10), Cibotium barometz J. Smith (8)	Replenishing qi and yin energy
Bb	Angelica gigas (7.5, chlorogenic acid, nodakenin), *Rehmannia glutinosa* (7.5, 5-HMF), Cnidium officinale Makino (7.5), *Spatholobus suberectus Dunn* (12)	Increasing erythropoietic activity and blood function
Bc	Prunus persica (7.5, amygdaline), Paeonia lactiflora Pall (7.5, albiflorin, paeoniflorin), *Carthamus tinctorius* (7.5), Lumbricidae (5), *Salvia miltiorrhiza* (12)	Improving blood circulation
Bd	*Uncaria rhynchophylla* (12), Pueraria lobate Ohwi (8, puerarin), *Crassostrea gigas* (12), *Albizia julibrissin Durazz* (12), Chaenomeles sinensis Koebhne (8)	Relieving pain and regulating yang energy
Be	*Panax ginseng C. A.Meyer* (4, ginsenoside Rg1, Rb1), *Angelica gigas* (7.5), Paeonia lactiflora Pall (7.5, albiflorin, paeoniflorin), *Crassostrea gigas* (8)	

^a^Number in parenthesis: grams in preparing each decoction

^b^Underlined in parenthesis are chemical ingredients which were identified by HPLC analysis

### HPLC analysis

Herbal extracts of BGJTD and of Ba, Bb, Bc, Bd, and Be subgroups were purified by filtration through 0.45 μm PVDF membrane, and standard solutions such as amygdalin, chlorogenic acid, ginsenosides Rg1 and Rb1, nodakenin, albiflorin, paeoniflorin, puerarin, and 5-HMF were diluted with ultra--purified water to a range of 50 – 500 ppm. The herbal extracts and standard solutions were analyzed by LC20A Series HPLC system (Shimadzu, Kyoto, Japan). The analysis system includes an Agilent eclipse plus C18 (250x4.6 mm) with linear elution with acetonitrile gradient at a flow rate of 1.0 ml/min. Sample aliquots (10 μl) were injected to the column, which was set to 40 °C. UV values for detection were 215 nm for amygdalin, 203 nm for Rg1 and Rb1, 230 nm for puerarin, albiflorin and paeniflorin, 280 nm for 5-HMF and chlorogenic acid, and 330 nm for nodakenin.

### Experimental animals

Sprague-Dawley rats (male, 200–250 g, Samtago, Seoul, Korea) or Balb/c mice (male, 22–25 g, Samtago, Seoul, Korea) were maintained in an animal room with regulated temperature (22 °C), 60 % of humidity, and 12-h light/dark cycle (light on 7 am to 7 pm). They were allowed to eat commercial pellet chow (Samyang Co., Seoul, Korea) and drink water ad libitum. All procedures were in strict accordance with the NIH guide for the care and use of laboratory animals and approved by the Committee on Use of Live Animals for Teaching and Research at Daejeon University (Daejeon, Korea).

### Animal surgery

For an experiment examining the effects of BGJTD in taxol-treated sciatic nerve, animals were randomly assigned into dimethysulfoxide (DMSO) vehicle-injected group (Veh), taxol-injected group (Taxol), and taxol plus BGJTD treated group (Taxol + BGJTD). Sciatic nerves and DRG at levels of lumbar 4 and 5 were prepared from individual animals and used for immunofluorescence staining and western blot analysis. Rats were anesthetized by injecting intraperitoneally a single dose of a mixture of ketamine (80 mg/kg) and xylazine (5 mg/kg). Taxol (3.6 mg/ml of 5 % DMSO; 1.25 mg/kg of body weight) and equivalent volume of DMSO as vehicle control were slowly injected by using a Hamilton syringe (10 μl-model, Innovative Labor System GmbH, Germany) into the sciatic nerve pre-exposed on the middle thigh. A similar procedure of local injection of taxol into the peripheral nerves were used in previous studies to examine regenerative or degenerative effects of taxol on the nerve [15, 16]. In order to induce acute effect on the microtubule integrity within axon of the peripheral nerve, we injected taxol with higher dosage than those used to investigate the regenerative responses of peripheral and spinal cord axons after injury [10, 16]. Twenty four hours later, BGJTD (400 mg/kg) was administered orally and was supplemented on a daily basis for 4 more days. Animals were sacrificed at day 7.

In an experiment investigating the effects of BGJTD on regenerative responses of injured axons, animals were randomly assigned into three groups: sciatic nerve injury plus BGJTD treatment (SNI + BGJTD), sciatic nerve injury plus saline (SNI + Sal), and non-treatment (Intact). To induce nerve injury, the sciatic nerve was exposed from the middle thigh and crush injury was given by holding the nerve with the forceps for 30 s twice as described previously [17]. BGJTD or saline was administered orally 24 h later and supplemented on a daily basis for 4 more days. Animals were sacrificed at day 7, and sciatic nerve or DRG sections (20 μm thickness) were prepared.

### Retrograde tracing of DRG neurons

For retrograde tracing of the sensory neurons in the DRG, the sciatic nerve from anesthetized rats with ketamine and xylazine was exposed and, immediately after nerve injury, fluorescent lipophilic carbocyanine dye l,l'-dioctodecyl-3,3,3',3'tetramethylindocarbocyanine perchlorate (DiI; 5 μl of 0.5 % in DMSO) was applied with a micropipette to the area 10 mm distal to the injury site. After suturing the incision, animals were recovered from the narcosis and returned to their cages. BGJTD or saline was administered orally 24 h later and supplemented on a daily basis for 4 more days, and the animals were sacrificed at day 7. The total number of DiI-labeled neurons from three nonconsecutive sections (20 μm thick) per animal was counted, and the mean percentage of labeled cells was compared between BGJTD and saline administered groups.

### Primary Schwann cell and DRG sensory neuron culture

For Schwann cell culture, intact sciatic nerve (1 cm length) was dissected from the rats and dissociated with 0.5 mg/ml type XI collagenase (Sigma, USA) in BME for 90 min at 37 °C. DRG neurons at lumbar 4–5 in adult rats were prepared 3 days after sciatic nerve injury, dissected, and dissociated similarly for neuron culture. After washing twice with BME, cells were treated with 5 μg/ml type SII trypsin for 15 min and followed by inhibition reaction for 5 min in 50 μg/ml of soybean trypsin inhibitor, 0.5 mM EDTA, and 20 μg/ml of DNase I. Cells (1 × 10^4^ cells per 24-well plate) were plated onto 12 mm coverslips (Bellco, Glass Inc. Vineland, USA) precoated with 0.01 % poly-L-ornithine (Sigma, St. Louis, MO) and laminin (0.02 mg/ml, Collaborative Research, Bedford, MA). Cells were cultured for 12 h, changed to BME containing 10 % serum (5 % fetal bovine serum plus 5 % horse serum) and 2 mM glutamine and 1 % penicillin-streptomycin. Cells were treated with herbal drugs (0.5 mg/ml) and incubated for 48 h before the harvest for immunofluorescence staining.

For DRG sensory neuron and Schwann cell co-culture, Schwann cells (1 × 10^4^ cells per 12 mm coverslip in 24-well plate) were cultured for 24 h and DRG sensory neurons (1.5 × 10^2^ cells) were added. The coculture was maintained in 500 μl of BME medium supplemented with 10 % serum and treated with taxol (0.01 mg/ml) alone or together with BGJTD (0.5 mg/ml) for 48 h prior to cell harvest. Equivalent volume of DMSO and saline as vehicle controls for taxol and BGJTD was treated, respectively. After immunofluorescence staining with antibodies against neurofilament-200 (NF-200) (1:400, Sigma), βIII-tubulin (TUJ1, 1:400, Covance), S100β (H-56, 1:400, Santa Cruz Biotech.), and caspase 3 (1:500, Cell Signaling), digital images of neuronal process were captured and transferred to the Adobe Photoshop Program. The length of neurite processes exhibiting clear outgrowth (longer than cell diameter) from the cell body was analyzed by i-Solution software program (Image and Microscope Technology, Burnaby, Canada). Mean neurite length was determined by analyzing at least 30 sensory neurons which were randomly selected in each experiment.

### Western blot analysis

Nerve segment was suspended in 100–200 μl of triton lysis buffer (20 mM Tris, pH 7.4, 137 mM NaCl, 25 mM β-glycerophosphate, pH 7.14, 2 mM sodium pyrophosphate, 2 mM EDTA, 1 mM Na_3_VO_4_, 1 % Triton X-100, 10 % glycerol, 5 μg/ml leupeptin, 5 μg/ml aprotinin, 3 μM benzamidine, 0.5 mM DTT, 1 mM PMSF) and was sonicated. The supernatant was taken after centrifugation at 12,000 rpm for 10 min at 4 °C. Protein (15 μg) was used for SDS-polyacrylamide gel electrophoresis and immunoblotting with anti-phospho-Erk1/2 antibody (1:4000, Cell Signaling), anti-Cdc2 antibody, anti-phospho-vimentin antibody (1:2,000, MBL) that binds specifically to phosphorylated vimentin (serine 55) by Cdc2 kinase [18], and horseradish peroxidase (HRP)-conjugated secondary antibodies (1:1000; goat anti-rabbit; Santa Cruz, or sheep anti-mouse; Amersham Biosciences, Buckinghamshire, UK). Densitometric analysis of protein bands in the X-ray film was determined using the i-Solution software (Image & Microscope Technology).

### Immunofluorescence staining

Samples were fixed with 4 % paraformaldehyde and 4 % sucrose in PBS at room temperature for 40 min, permeablized with 0.5 % nonidet P-40 in PBS, and blocked with 2.5 % horse serum and 2.5 % bovine serum albumin for 16 h at room temperature. Staining procedure was performed by incubating with primary antibodies raised against Cdc2 (1:100, Santa Cruz Biotech.), vimentin (1:1,000, Chemicon, Temecula, USA), phospho-vimentin (1:400, MBL), NF-200 (1:400, Sigma), βIII-tubulin (TUJ1, 1:400, Cavance), S100β (1:400, Santa Cruz Biotech.), phospho-Erk1/2 (1:400, Cell Signaling), and GAP-43 (1:400, Santa Cruz Biotech.) followed by fluorescein-goat antimouse (1:400, Molecular probes, Eugene, OR, USA) or rhodamine-goat anti-rabbit secondary antibodies (1:400, Invitrogen, Carlsbad, CA, USA) in 2.5 % horse serum and 2.5 % bovine serum albumin for 90 min at room temperature. Cellular nuclei were stained with 2.5 μg/ml of Hoechst dye 33258 (bis-benzimide; Sigma) for 10 min before the final washing with 0.1 % Triton X-100 in PBS, and the sections or cells were cover-slipped with gelatin mount medium. Samples were viewed with a Nikon fluorescence microscope, and the images were captured by Nikon camera. The merged images were produced using layer-blending mode options of the Adobe Photoshop. For quantitative analysis of image data, average signals from 4 nonconsecutive sections per each animal were compared among experimental groups.

### Hot plate test

Sciatic nerves of Balb/c mice (male, 22–24 g) were exposed on the middle thigh and taxol (1.25 mg/kg) or equivalent volume of DMSO vehicle was focally injected into the sciatic nerve. Twenty four hours later, herbal decoction (400 mg/kg) or saline was administered orally and was supplemented on a daily basis for 4 more days. On day 7 after taxol injection, animals were subjected to thermal sensitivity test on a hot plate. Animals were adapted for 10 min on the surface on a hot plate adjusted to 30 °C, and immediately after, were placed for 30 s on the plate adjusted to 50 °C. The latency to the lifting response of the hind paw and the number of withdrawal frequency were determined by analyzing real-time images of animal movement which were captured by a digital camera.

### Statistical analysis

Data were presented as mean ± standard error of mean (SEM). The mean number data in individual groups were compared by the Student’s *t*-test or one-way ANOVA followed by Tukey test post-hoc analysis (SPSS computer software version 21.0), and statistically significant differences were reported as *P < 0.05, **P < 0.01, ***P < 0.001.

## Results

**Fig. 1:**
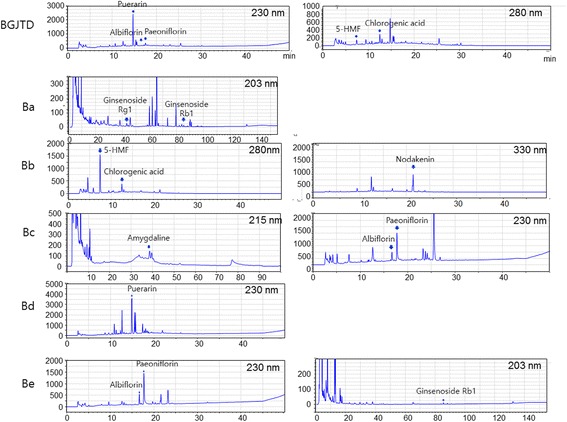
HPLC profile of BGJTD and its subgroup extracts. Peaks that coincide with standard chemical solutions are marked by vertical arrows, and UV values applied to detect peaks are indicated in the figure

To validate the chemical profile of herbal extracts, BGJTD and 5 subgroups, we carried out HPLC analysis for chemical ingredients of herbal drugs. BGJTD and 5 subgroup extracts showed the peak profiles in the chromatogram that coincide with those of standard chemical solutions (Fig. 1). Specifically, HPLC profiles of BGJTD identified puerarin in Pueraria lobate Ohwi, albiflorin in Paeonia lactiflora pall, paeoniflorin in Paeonia lactiflora pall, 5-HMF in Rehmannia glutinosa, and chlorogenic acid in Angelica gigas. Other ingradients in Ba to Be subgroup decoctions are indicated in Table 2.

**Fig. 2:**
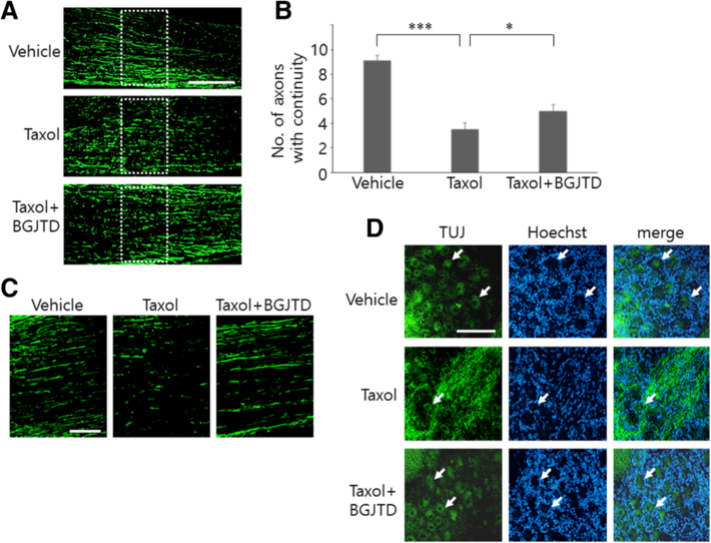
Morphological changes of sciatic nerve axons and DRG neurons after taxol injection and BGJTD treatments. Sciatic nerve was exposed and treated with DMSO vehicle, taxol, or taxol plus BGJTD, and longitudinal sections of the nerve at the injected location (**a**, **c**) and DRG at lumbar level 5 (**d**) were used for immunofluorescence staining for NF-200 and βIII-tubulin. The number of axons whose length is longer than 100 μm, as illustrated with dotted rectangles in (**a**), was counted from the images, averaged from 3 nonconsecutive sections and compared among 3 experimental groups. Quantitative data are shown in (**b**). Soma DRG neurons was marked by arrows in (**d**). *p < 0.05, ***p < 0.001 (One-way ANOVA, number of animals = 4). Scale bars in (**a**, **c**, **d**): 100 μm

To investigate whether taxol and BGJTD influence on structural integrity of the peripheral nerve, sciatic nerve axons were visualized by immunofluorescence staining using anti-NF-200 antibody as axon marker. Comparison among experimental groups showed that the mean length of individual axons in the longitudinal nerve sections were significantly decreased by taxol injection indicating their fragmentation and then improved by BGJTD administration (Fig. 2a, b). Given that taxol stabilizes mictotubule assembly, we further investigated axonal morphology by immunostaining with neuron-specific β-III tubulin antibody (TUJ-1). Staining pattern of the sciatic nerve axons, as seen by elongated fibers in DMSO vehicle control, was disintegrated by taxol injection, which was then largely recovered in its integrity by BGJTD administration (Fig. 2c). Examination of neurons in the DRG at lumbar level 5, where the cell bodies of sciatic nerve sensory axons reside, showed structural disruption of the neurons by taxol injection into the nerve. However, in animals administered with BGJTD, some, but not all, of neurons showed the morphology that is similar to those in the control animals (arrows in Fig. 2d).

**Fig. 3:**
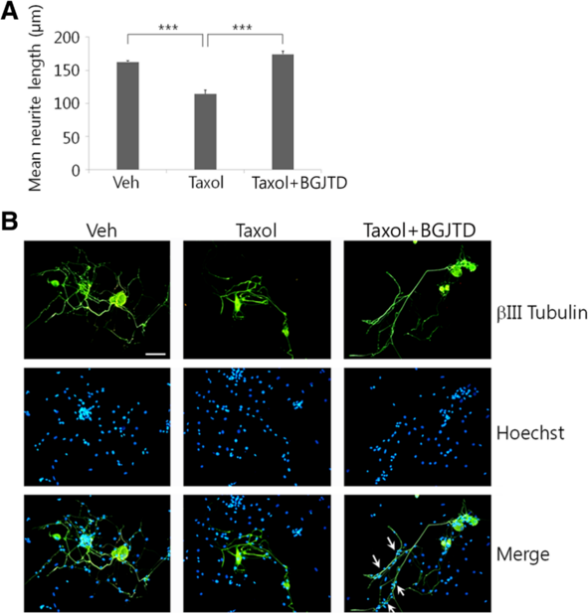
Effects of BGJTD on the neurite outgrowth of DRG neurons. **a** Quantitative comparison of neurite outgrowth. **b** Representative images of DRG neurons co-cultured with Schwann cells. The neurites were visualized by immunostaining with anti-βIII tubulin antibody (green), and the distribution of co-cultured Schwann cells were identified by Hoechst nuclear staining (blue). In (**a**), neurite length was determined by analyzing more than 20 neurons in 7–10 random microscopic fields, averaged 4 independent experiments, and compared among experimental groups. Error bars denote standard error of mean (SEM). ***p < 0.001 (One-way ANOVA, number of independent experiments = 4). Scale bar: 50 μm

Next, we examined the effects of taxol and BGJTD treatments on the neurite outgrowth of cultured DRG neurons. Taxol injection significantly reduced neurite length, but cotreatment with BGJTD improved neurite extension similar to vehicle-treated control group (Fig. 3a). Examination of DRG neurons merged with Hoechst-stained nuclear images revealed that many of non-neuronal cell nuclei in the culture were highly colocalized with the neurite processes, which was observed most clearly in a group treated with taxol and BGJTD (arrows in Fig. 3b).

**Fig. 4:**
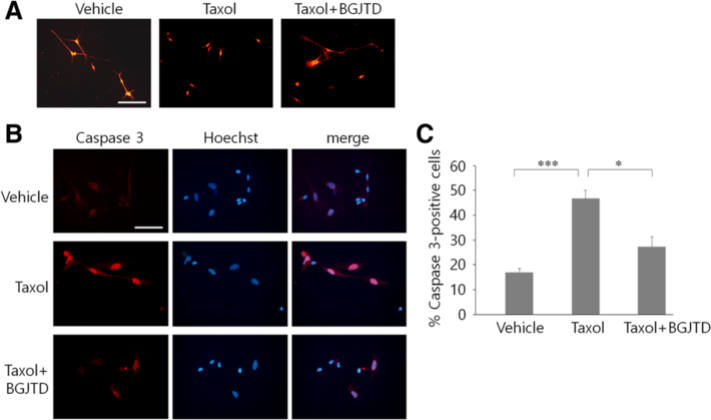
Regulation of Schwann cell survival by BGJTD treatment. Schwann cells prepared from intact sciatic nerve were treated with DMSO vehicle, taxol, and taxol plus BGJTD. Fixed cells were used for immunostaining with anti-S100β antibody (**a**) and anti-caspase 3 antibody (**b**). Cells were also stained with Hoechst33258 to visualize individual nuclei (blue). **a** Morphological comparison of S100β-stained Schwann cells after different treatments. **b** Immunofluorescence staining of caspase 3 in cultured Schwann cells. **c** Quantitative comparison of caspase 3-positive cells in cultured cells after different treatments. A percentage of caspase 3-positive cells from randomly selected microscopic fields (more than 5 fields) were determined from each experiment. *p < 0.05, ***p < 0.001 (One-way ANOVA, number of independent experiments = 4). Scale bars in (**a**-**c**): 100 μm

Given that taxol interferes dynamic regulation of microtubule assembly, Schwann cells in the nerve might be affected adversely by taxol. Some Schwann cells in the control group showed thin, elongated morphology having physical contact with other cells. After taxol treatment, cells became more spherical and smaller (Fig. 4a), but co-treatment with BGJTD reverted them into an elongated morphology, which was similarly observed in control Schwann cells. To determine the involvement of taxol and BGJTD in cell death regulation, levels of caspase 3 as a marker of apoptotic cell death were analyzed in cultured Schwann cells. Caspase 3 signals were clearly induced by taxol, but decreased to a large extent by BGJTD treatment (Fig. 4b, c).

**Fig. 5:**
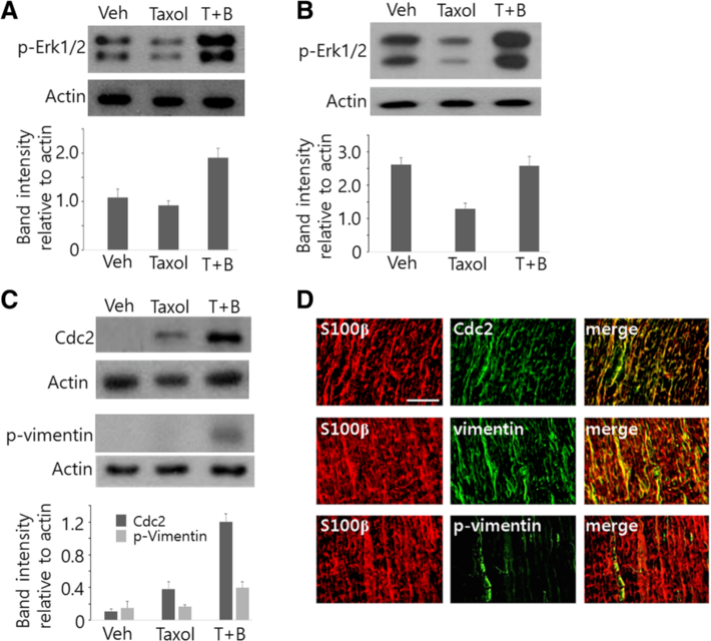
Effects of taxol and BGJTD treatments on Erk1/2 and Cdc2 activity in the sciatic nerve. Western blot analysis of phospho-Erk1/2 in the sciatic nerves (**a**) and in the DRG (**b**) after taxol injection and BGJTD treatments (T + B). **c** Western blot analysis of Cdc2 and phospho-vimentin in the sciatic nerves treated with DMSO vehicle, taxol, and taxol plus BGJTD. **d** Representative images showing Cdc2 and phospho-vimentin signals in S100β-labeled Schwann cells. Longitudinal sections from distal portion of the nerve treated with taxol and BGJTD were used in this experiment. Western blotting images in (**a**-**c**) are the representatives from 3 independent experiments. Quantitative comparison of mean band intensity for target proteins relative to actin control in western blotting are shown in lower panels in (**a**-**c**). Scale bars in (**d**): 100 μm

To investigate that BGJTD and taxol treatments alter neural cell activity, we examined Erk1/2 activation in the sciatic nerve and DRG. Phospho-Erk1/2 levels was not altered by taxol alone, but increased by BGJTD administration (Fig. 5a). In the DRG, phospho-Erk1/2 levels were decreased by taxol, and then increased by BGJTD administration (Fig. 5b). As another indicator of nerve activation, we examined induction of Cdc2 kinase and its phosphorylation of vimentin intermediate filament protein as a substrate, which is known to be induced strongly but transiently from Schwann cells in the injured peripheral nerve [19, 20]. Cdc2 was induced in the sciatic nerve by taxol injection, and BGJTD treatment upregulated Cdc2 with concomitant induction in phospho-vimentin (Fig. 5c). Immunohistochemical analysis for the sciatic nerve showed that both Cdc2 and phospho-vimentin were detected together in S100β-labeled Schwann cells (Fig. 5d).

**Fig. 6:**
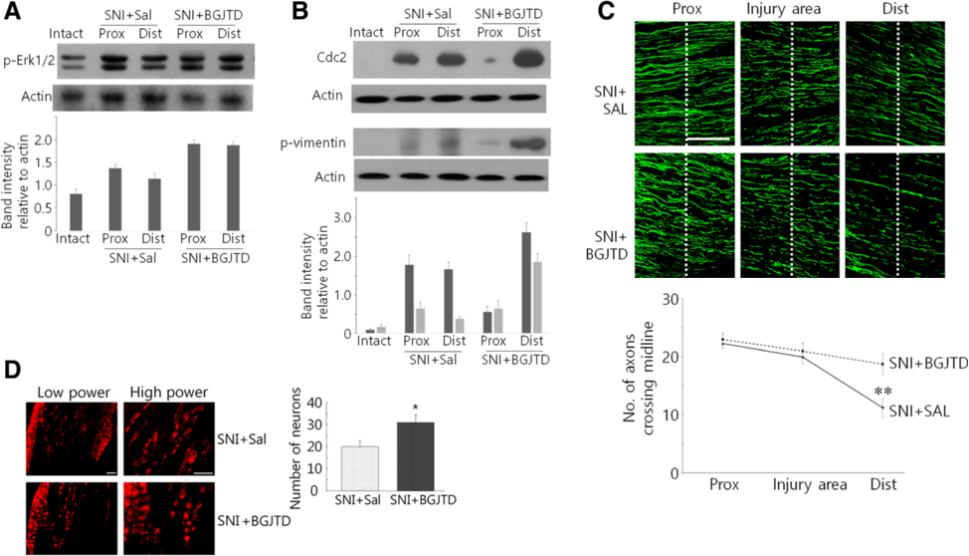
Effects of BGJTD treatment on the axonal regeneration after nerve injury. **a** Western blot analysis of phospho-Erk1/2 after sciatic nerve injury (SNI) and BGJTD treatment. **b** Western blot analysis of Cdc2 and phospho-vimentin in the sciatic nerve after injury (SNI) and BGJTD treatment. In (**a**) and (**b**), western blotting images are the representatives from 2–3 independent experiments. Quantitation of protein band intensity relative to actin control are shown in lower panels. **c** Distribution of axons in the sciatic nerve after crush injury. Axons were visualized in the proximal, injury, and distal stumps (6 mm each) in the longitudinal sections by immunofluorescence staining for NF-200. (Lower) Number of axons crossing midline (dotted) are counted from each images, averaged from nonconsecutive sections from each animal, and compared between experimental groups (One-way ANOVA, **p < 0.01, number of animals = 4). **d** Retrograde tracing of DiI-labeled sensory neurons in the DRG (left). Quantitative comparison of DiI-labeled DRG neurons between saline-and BGJTD-treated groups (right).*p < 0.05 (Student’s *t*-test, number of independent experiments = 4). Scale bars in (**c**, **d**): 100 μm

We further investigated whether BGJTD was beneficiary to axonal regeneration of the sciatic nerve after crush injury. Phospho-Erk1/2 levels were increased by crush injury and further upregulated by BGJTD treatment (Fig. 6a). Cdc2 was clearly induced in both proximal and distal portions of the sciatic nerves at day 7 after injury (Fig. 6b). Then the treatment of BGJTD suppressed Cdc2 production almost exclusively in the proximal stump while further increasing in the distal stump. Phospho-vimentin was weakly detected in the injured sciatic nerves but greatly increased in the distal portion of the nerve after BGJTD treatment. Immunofluorescence staining of the nerve fibers revealed that axonal elongation to the distal portion of the nerve was restricted to a certain extent when measured 3 days after injury; however, axon extension was much improved by BGJTD treatment (Fig. 6c). For quantitative analysis of axon regeneration by BGJTD, DRG neurons were retrogradely labeled by fluorescence dye DiI. DiI-labeled neurons, as an indicator of regenerating sensory axons, were clearly observed in many of DRG neurons, and the total number of labeled neurons was significantly higher in BGJTD-treated group than control group (Fig. 6d).

**Fig. 7:**
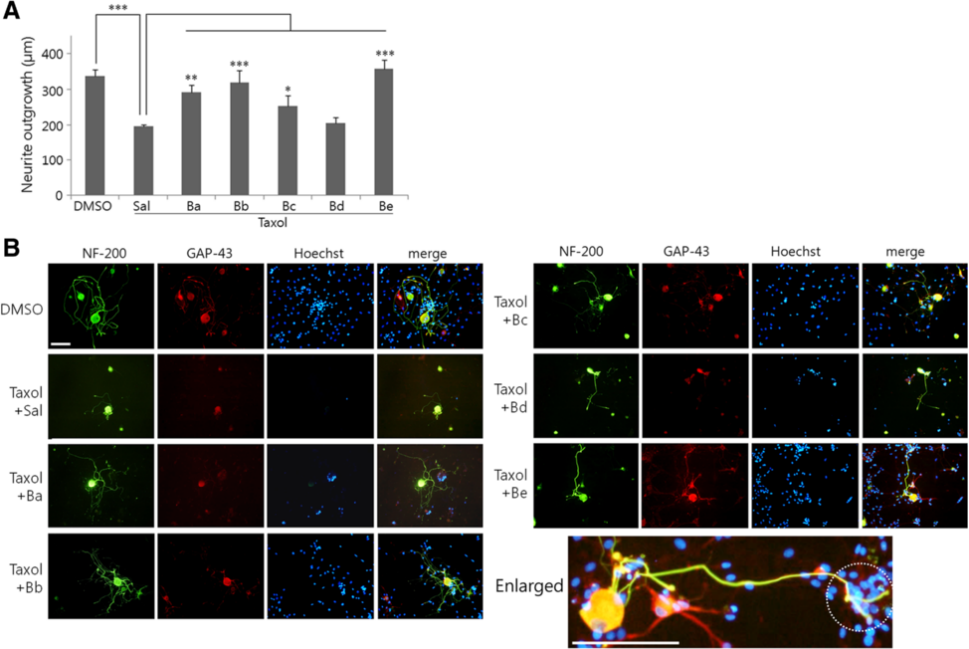
Effects of BGJT subgroup decoctions on neurite outgrowth of taxol-treated DRG sensory neurons. Six days after taxol or DMSO vehicle injection in vivo into the sciatic nerve, DRG neurons were cultured in the presence of individual decoctions Ba to Be (0.5 mg/ml) for 48 h and harvested for immunostaining. **a** Comparison of neurite length among experimental groups. **b** Representative images of cultured cells after immunofluorescence staining with NF-200 (Green) and GAP-43 (red). Cell nuclei were visualized by nuclear staining with Hoechst 33258 (blue). In (**a**), neurite length was determined by analyzing more than 20 neurons in 7–10 random microscopic fields, averaged 4 independent experiments, and compared among experimental groups. Error bars in (**a**) denote standard error of mean (SEM). *P < 0.05, **P < 0.01, ***p < 0.001 (One-way ANOVA, *N* = 4). Scale bars in (**b**): 100 μm

**Fig. 8:**
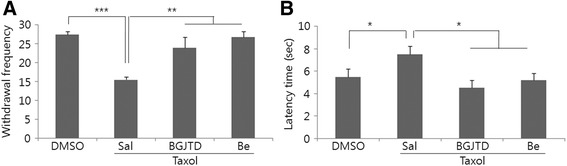
Effects of taxol, BGJTD, and Be drugs on the heat sensitivity. Mice were given taxol followed by oral administration of BGJTD or Be, and were subjected to measure the lifting frequency of the hind paws (**a**) and the latency period after placing on the hot plate (**b**). Error bars in (**a**) denote standard error of mean (SEM). *P < 0.05, **P < 0.01, ***p < 0.001 (One-way ANOVA, number of animals =4)

BGJTD is a decoction composed of as many as 18 different herbal drugs. As an initial step to sort out the active components in its efficacy on the nerve repair, we divided them into 4 different groups (i.e., Ba, Bb, Bc, and Bd) based on the description of the traditional medicine (Table 2). After treating individual herbal drugs to cultured DRG neurons, we selected the most effective herbal drug from each group in inducing neurite outgrowth, and prepared the fifth herbal drug group, termed ‘Be’ (Table 2) (manuscript in preparation). We then treated each decoction to taxol-treated DRG neurons, and compared the neurite outgrowth. In vivo taxol injection suppressed neurite outgrowth and abrogated Schwann cell survival to a large extent as indicated by Hoechst nuclear staining (Fig. 7a, b). While the recovering effects of drug treatment on neurite outgrowth were variable, Be decoction was the most effective to enhance neurite outgrowth. It was also noted that the number of Hoechst-stained nuclei in Be was higher than other drugs and the elongated neurite was in close contact with many nuclei particularly at the tip (marked with a dotted circle in the enlarged image in Fig. 7b). In Be group, GAP-43 protein signals were not only observed in the cell body as other groups, but also were seen clearly in the elongating neurites. We further investigated the behavioral effect of BGJTD and Be extract on heat sensitivity. Withdrawal frequency and latency time in lifting the hind legs from the hot surface were significantly impaired by taxol administration, but were improved by BGJTD or Be treatment (Fig. 8a, b).

## Discussion

The objective of the present study was to investigate whether BGJTD was effective for protecting the peripheral nerves from damage caused by taxol injection and physical injury. Taxol generated structural disruption of the nerve, and hampered Schwann cell survival adversely. Morphological and biochemical analyses of the sciatic nerve and DRG tissue revealed that taxol and crush injury generated nerve damage in a similar way. Importantly, these pathological injuries were largely recovered by BGJTD treatment. By sorting out the herbal components of BGJTD in terms of their activity to enhance neurite outgrowth, we selected the active herbal components, and a decoction Be from selected drugs was as effective as BGJTD in facilitating neurite outgrowth and responding to heat stimulation.

Since the initial discovery and pharmacological characterization, taxol has been used as a chemotherapeutic drug. However, together with several other anticancer drugs such as platinum compounds, taxanes, vinka alkaloids, bortezomib, and thalidomide, a large portion of patients given taxol therapy showed neuropathy accompanying severe pain [21, 22]. Considering stabilization effect of taxol on microtubule assembly, axonal cytoskeletal structures mainly composed of microtubule assembly might be damaged by taxol therapy. Studies with animals and in vitro culture showed that taxol caused inhibition of neurite outgrowth of cultured neurons, disrupted myelin structure, and retarded conduction of action potential [7–9].

After peripheral nerve injury, neuropathy can occur during repair process [2, 23]. BGJTD has been used for the treatment of neuropathic pain and regulation of blood glucose levels in diabetic patients in the clinical traditional medicine, implicating the possible ameliorating effects of BGJTD on diabetic or anticancer-related neuropathy [11, 12]. To understand biological basis of BGJTD action, we performed experiment using two models of peripheral nerve injury, and reached following conclusions; first, we found that taxol injection not only inhibited neurite outgrowth of cultured DRG neurons but also induced morphological abnormalities resulting in apoptotic death of primary Schwann cells. Taxol may have a detrimental effect on axonal regrowth by inhibiting Schwann cell interaction with axons [24]. Furthermore, functional degeneration of Schwann cells by taxol injection may prevent axon myelination and lead to morphometric alterations in action potential propagation [25, 26]. Secondly, direct effects of taxol on the sciatic nerve axons were most clearly seen by immunohistochemical analysis in which βIII tubulin-labeled axons were largely degenerated by taxol injection. The structure of βIII tubulin-labeled DRG neurons at lumbar 5 was severely disintegrated by taxol injection, and phospho-Erk1/2 signals in DRG neurons were largely decreased by taxol. Previous studies have shown that phospho-Erk1/2 is induced in the peripheral nerve after injury, and can be transported retrogradely into the cell body where it leads to expression of target protein involved in axonal repair [27–29]. Here, taxol that may cause structural derangement from axon to soma could disrupt retrograde transport of Erk1/2 signaling, as has been shown by retarded transport of tracer in taxol-treated nerve [30]. Finally, BGJTD administration produced regenerative responses in the peripheral nerve. Signal intensity of apoptotic marker protein caspase 3 in taxol-treated Schwann cells was reduced by BGJTD, and Cdc2 activity, which was induced in the sciatic nerve after taxol injection or crush injury, was upregulated in the distal portion of the nerve. Moreover, neurite extension in cultured DRG neurons and axonal staining of in vivo sciatic nerves were improved in the BGJTD-treated groups, compared to corresponding taxol-treated ones.

We have reported that vimentin phosphorylated by Cdc2 kinase is induced in Schwann cells from the injured nerve and involved in regenerative responses [19, 20]. Here, Cdc2 and phospho-vimentin were detected from taxol-injected sciatic nerve, and interestingly, their levels were further upregulated by BGJTD treatment. Given that Cdc2 activation in Schwann cells is responsible for axonal regeneration, BGJTD-mediated Cdc2 induction in Schwann cells may facilitate repair process in the taxol-treated nerves. Indeed, neurite outgrowth in the taxol-treated DRG neurons was recovered by BGJTD to the level of control ones, and in vivo axon morphology, as identified by βIII-tubulin staining, was clearly improved by BGJTD. The protective effect of BGJTD was also shown in Schwann cells in which caspase 3 signals induced by taxol was largely decreased by BGJTD treatment. Thus, protective activity of BGJTD in both individual axons and Schwann cells may improve nerve repair, as demonstrated by increases in axon staining in the distal nerve stump and retrograde labeling of DRG sensory neurons (Fig. 6).

Our data showing the induction of Cdc2 and phospho-vimentin levels by BGJTD suggest possible molecular basis on how BGJTD acts on neuropathy. The BGJTD treatment upregulated Cdc2 levels and its phosphorylation of vimentin in taxol-injected nerves and the distal stump in the injured nerve. It was previously reported that Schwann cell activation in terms of Cdc2-vimentin pathway is beneficiary to promote axonal regeneration of the peripheral nerve [20]. After peripheral nerve injury, axons in the distal stump degenerate and regrow from the proximal end, and in this process, Schwann cells are actively involved in eliminating degenerating debris and guiding the growth cones at the front [24]. Molecular factors that are expressed from activated Schwann cells including growth factors and anti-inflammatory cytokines may play a role in alleviating peripheral nerve neuropathies [31, 32]. Timely interaction of Schwann cells with axons is important for recovering distal portion of injured nerve with minimizing inflammation [33]. It was reported that activation of Cdc2-vimentin pathway in Schwann cells was linked to activation of membrane intergrin, which is further involved in intercellular communication with regrowing axons [20, 24]. Whether BGJTD therapy is functionally associated with Schwann cell communication via integrin remains to be determined.

BGJTD is a decoction composed of 18 different herbal drugs and has been used in Asian medicine to treat neuropathy caused by diabetes and anticancer therapy [11]. A decoction shengmai san, which is used for the treatment of cardiovascular disorders in traditional medicine, shares the herbal component Ginseng radix with BGJTD and was reported to be involved in promoting regenerative responses after spinal cord injury [34]. Ginsenoside components such as Rg1 and Re were reported to facilitate axonal regeneration after peripheral nerve injury, or neuronal survival [35, 36], and ginsenoside Re was shown to activate Schwann cells in the injured nerve thereby promoting axonal regeneration [37]. However, given that BGJTD contains diverse herbal components, it is difficult to determine whether the efficacy of BGJTD is related to combinatorial activity or due to some specific components. Here, as an initial step to screen the possible active herbal components, we categorized 18 individual drugs into 4 groups based on description of the traditional medicine, and selected the most active components inducing neurite outgrowth of cultured neurons; a similar procedure was applied previously to search herbal drugs for therapeutic application to neurodegenerative diseases [38]. Our data showed that Be decoction composed of 4 active herbal drugs is as effective as BGJTD in inducing heat sensitivity of the hind paws. Further studies are critical to determine whether Be extract is comparable to BGJTD in regulating regenerative responses in association with peripheral nerve neuropathy.

## Conclusions

In conclusion, our data showed that BGJTD, which is used clinically in the traditional Asian medicine, has a protective effect on neuropathic insults such as taxol injection or crush injury. By using in vitro screening procedure, we have selected some active herbal components, of which decoction displayed the efficacy of reinducing the optimal levels of heat sensitivity in taxol-treated animals. Accordingly, our data provide the biological basis of BGJDT on the treatment of the peripheral nerve neuropathy, and further implicate that some of the traditional medicinal descriptions on the efficacy of the herbal drugs may be applicable to the scientific approaches to identifying the active ingredients for the purpose of the therapeutic strategy.

## Abbreviations

BGJTD: Bogijetong decoction
SNI: Sciatic nerve injury
DRG: Dorsal root ganglion
Cdc2: Cell division cycle 2
GAP-43: Growth-associated protein 43
